# XGSleeve: detecting sleeve incidents in well completion by using XGBoost classifier

**DOI:** 10.3389/frai.2023.1243584

**Published:** 2023-09-13

**Authors:** Sahand Somi, Sheikh Jubair, David Cooper, Peng Wang

**Affiliations:** ^1^Advanced Technology, Alberta Machine Intelligence Institute, Edmonton, AB, Canada; ^2^DevOps, Kobold Completions Inc., Calgary, AB, Canada

**Keywords:** oil and gas, well completion, sliding sleeves, time series classification, signal processing, XGBoost, hidden Markov model

## Abstract

The *sliding sleeve* holds a pivotal role in regulating fluid flow during hydraulic fracturing within shale oil extraction processes. However, concerns persist surrounding its reliability due to repeated attempts at opening the sleeve, resulting in process inefficiencies. While downhole cameras can verify sleeve states, their high cost poses a limitation. This study proposes an alternative approach, leveraging downhole data analysis for sleeve incident detection in lieu of cameras. This study introduces “XGSleeve,” a novel machine-learning methodology. XGSleeve amalgamates hidden Markov model-based clustering with the XGBoost model, offering robust identification of sleeve incidents. This method serves as an operator-centric tool, addressing the domains of oil and gas, well completion, sliding sleeves, time series classification, signal processing, XGBoost, and hidden Markov models. The XGSleeve model exhibits a commendable 86% precision in detecting sleeve incidents. This outcome significantly curtails the need for multiple sleeve open-close attempts, thereby enhancing operational efficiency and safety. The successful implementation of the XGSleeve model rectifies existing limitations in sleeve incident detection, consequently fostering optimization, safety, and resilience within the oil and gas sector. This innovation further underscores the potential for data-driven decision-making in the industry. The XGSleeve model represents a groundbreaking advancement in sleeve incident detection, demonstrating the potential for broader integration of AI and machine learning in oil and gas operations. As technology advances, such methodologies are poised to optimize processes, minimize environmental impact, and promote sustainable practices. Ultimately, the adoption of XGSleeve contributes to the enduring growth and responsible management of global oil and gas resources.

## 1. Background

Canada is a significant player in the global energy market, with its vast natural resources that allow it to be one of the world's largest energy producers and exporters (Canada Energy Center, 2022). The country's energy exports, particularly its oil and gas products, have contributed significantly to its economy (Canada Energy Center, 2022). According to the Canadian Energy Center, in 2020, Canada exported $63.8 billion worth of crude oil and $8.9 billion in natural gas (Canada Energy Center, 2022). This underscores the importance of the energy sector to the country's overall economic wellbeing (Canada Energy Center, 2022).

In recent decades, the Canadian energy sector has witnessed a notable advancement with the large-scale development of shale resources (Government of Canada, 2020). Shale resources encompass unconventional oil and gas reserves found deep underground within rock formations (Knaus et al., 2010). Unfortunately, the extraction processes associated with shale resources often suffer from inefficiencies, resulting in significant material waste that directly impacts Environmental, Social, and Corporate governance considerations known as ESG. To address ESG, the government has placed increased focus on reducing emissions and improving the efficiency of the oil and gas industry, aiming to mitigate its impact on climate change (Environment and Climate Change Canada, 2023).

To improve the efficiency of shale resource extraction, a new completion technology called coiled tubing-enabled fracturing sliding sleeve (CTFSS) was proposed in the last decade (Mahmud et al., 2020). This technology uses coiled tubing to carry a switch tool that opens or closes the sliding sleeve, allowing for oil well operations such as fracturing stimulation, selective exploitation, and closing the seal leakage layer, improving working efficiency. However, sliding sleeves are known to be unreliable, leading to incomplete operations and increased costs (Mahmud et al., 2020). The sleeve's unreliability often necessitates multiple attempts to open it or misleads operators into proceeding to the next step, like starting fracking prematurely. Consequently, this leads to a less efficient process and a significant waste of material, amounting to a value of 10,000 to 100,000 USD per sleeve. These issues are alarmingly common during the completion phase, requiring approximately twice the processing time for each fracking operation to be spent recycling the tool due to repeated attempts. Currently, the only option to capture downhole events during fracking operations is to deploy a camera inside the well, which is an exceedingly expensive solution.

In this project, we propose a new algorithm to collect downhole data, including wellhead vibration (Echo^©^ data), and analyze this information using a novel machine-learning approach that employs the XGBoost model to detect incidents where the sleeve fails to open. This approach will alert field operators when the sleeve is not open, preventing them from continuing the fracking process and ultimately leading to improved reliability and efficiency of sliding sleeves. Therefore, The contributions of this paper are twofold:

- We demonstrate how to successfully preprocess data and extract features that are meaningful for sleeve incidents.
- We propose a novel approach to collect and process Guidehawk^©^ and Echo^©^ data onsite and apply XGBoost model to provide real-time reporting of positive sleeve shifts. By increasing the certainty of sleeve shifts, this approach can significantly improve the completion process, leading to reduced time on location, increased productivity, and lower costs and environmental impact.

## 2. Related work

To replace the usage of expensive cameras in downhole a few researchers have suggested the use of downhole data to enhance the reliability of sleeve incidents and verify sleeve openings (Daniels and Williams, 2001). Utilizing downhole data offers a straightforward and cost-effective solution to enhance the dependability of sliding sleeve operations. Nonetheless, the tools employed to acquire this data are incapable of withstanding jarring loads (Daniels and Williams, 2001). To address this challenge, Welling et al. (2007) suggested that data analysis could be conducted upon retrieval to the surface. This analysis can primarily be utilized for troubleshooting any issues that may arise after the completion of the fracking process. However, detecting and troubleshooting sleeve incidents *in situ* provides even greater value. Therefore, Kenison et al. (2012) proposed measurements that are available at the surface during the operation, enabling the operator to control the shifting tool properly and identify any problems with the sleeve or shifting tool. The suggested measurements include coil tubing and annulus pressure, temperature, casing collar locator, and axial load, all of which can be collected by a tool in the Bottom Hole Assembly (BHA). However, the cost of these measurement tools is high, and the return on investment may not be substantial enough to encourage companies to incorporate them into their BHA.

Acknowledging these limitations, Kobold Completions Inc. (2023a) introduces a pioneering alternative that involves utilizing downhole data and capturing vibration signals on the surface using an Echo^©^. The installation and integration of the Echo and Guidehawk devices are remarkably straightforward, making them adaptable to various job settings. Both tools utilize familiar oil field sensors employed by other analogous devices (Kobold Completions Inc., 2023b). This approach provides a cost-effective and *in situ* solution when compared to using a tool in the BHA. Incorporating a conventional geophone, Echo can be easily fastened to the wellhead. Despite its specific usage in identifying acoustic signatures for fracking activity, these devices find application in monitoring neighboring wells for inter-well communication during fracks. The Echo device, being located on the surface, eliminates the need for advanced materials to protect the device and cable transmitting signals from the pressurized downhole environment. In addition, since sleeve openings result in sudden shocks, the Echo^©^ device can record the vibrations related to the shocks, providing valuable insights into sleeve performance and potential recycling issues. However, these devices themselves are not able to identify sleeve incidents—there is a critical need for a solution to take these time series data as input and identify the sleeve incidents in open or closed classes.

In this project, we propose a new method for identifying sleeve incidents using machine learning, both Echo^©^ and downhole data. Echo^©^ data will be collected from the Echo^©^ device, which captures vibration signals from the wellhead during operation, and a machine learning model will be used to confirm sleeve opening incidents based on these signals. In addition, Guidehawk^©^ data (downhole data) in the BHA will collect annular pressure (Pa), strain (N), shock (g), temperature (C), and torque (Nm) during operation. A machine learning model will be used to annotate opening incidents after and during operation, helping operators gain a better understanding of sleeve location during and after completion.

Due to the novel data set, this will be the first time leveraging machine learning on data from Echo^©^ and Guidehawk^©^ devices. However, the use of machine learning for incident detection in various industries has become increasingly popular due to its ability to identify patterns and anomalies in large data sets. In one example, a Convolutions Self-Attention Mechanism was proposed to detect rolling bearing faults (Ye et al., 2020). This model was able to outperform traditional machine learning models by 10% in precision and has shown promise in improving the efficiency and accuracy of predictive maintenance for industrial equipment. Another example is that an XGBoost Model was used for time series classification of solar flares by employing window-based feature extraction (McGuire et al., 2019). This model was able to successfully predict 75% of solar flares in the validation set, showcasing the potential of machine learning models in detecting solar flares and other space weather events. Esmael et al. (2012) proposed a new approach for time series classification problems such as failure detection. The proposed approach aims to combine the strengths of the Hidden Markov Model (HMM) in handling temporal data with other classification techniques to overcome their weaknesses. The approach is evaluated through a case study involving the classification of real drilling data generated by rig sensors, demonstrating its feasibility and effectiveness.

Furthermore, the application of machine learning in detecting ball bearing failure in offshore naturally flowing wells has also been explored (Nascimento et al., 2022). The proposed semi-supervised learning approach only considered nine features through a sliding window of 60 s and utilized a CNN-LSTM model. This model achieved an impressive 98% accuracy in the validation set, suggesting its potential in reducing maintenance costs and improving equipment reliability. In addition, machine learning has also been used to detect extreme events occurring in different time scales using hydrologic time series (Rolim and de Souza Filho, 2020). The authors proposed an HMM to detect regime shifts in weather data. This model is useful in providing early warning signs of weather-related incidents, allowing for better preparation and risk management.

Overall, these examples illustrate the potential of machine learning for detecting and preventing incidents in various industries while also highlighting the lack of data-driven processes in the well completion stage. In this project, our objective is to introduce a novel machine learning architecture to predict opening incidents in well completion by using vibration signals at the wellhead. To achieve our objective, we employed the Hidden Markov Model for clustering data points and the XGBoost model for classification.

## 3. Materials and methodology

This section provides a comprehensive overview of the Machine Learning (ML) models integrated into our project framework. Before delving into the development of these models, we extensively addressed the crucial data preprocessing stage. This stage involved a series of tasks, including gathering data from reliable sources, meticulously handling missing values and outliers through rigorous data cleaning procedures, and extracting meaningful features for time series data.

Upcoming subsections will offer in-depth insights into the precise methodologies utilized, along with the selected parameter configurations. These combined efforts significantly contribute to maintaining the integrity and reliability of our analysis.

### 3.1. Data

Kobold Completions Inc. (2023a), an industry leader in cutting-edge technology, specializes in providing more accurate, reliable, and efficient operations for sleeves. As the second largest manufacturer in Canada, their BHA is employed in various well completion processes. This project collects data from Kobold's proprietary technologies during fracking operations, including Echo^©^ surface monitoring and Guidehawk^©^ downhole memory tools. Echo^©^ facilitates real-time acoustic monitoring at the wellhead, delivering surface feedback on downhole events. It detects wellhead vibrations caused by down-hole activities, such as sleeve shifts, and presents the vibration signal in real time. The data is recorded as acceleration in meters per second squared and subsequently converted to units of gravitational acceleration on the Earth *g* = 9.81*ms*^−2^. On the other hand, Guidehawk^©^ is a downhole memory tool that logs multiple variables, including pressure for both coil and annular pressure (Pa), strain (N), shock (g), temperature (C), and torque (Nm). We collect Guidehawk^©^ and Echo^©^ data from various wells at different locations across Canada.

#### 3.1.1. Guidehawk^©^ data

**Figure 1:**
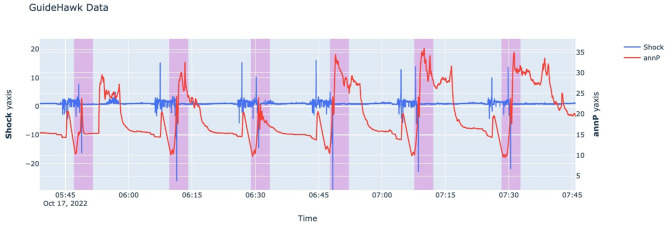
Guidehawk^©^ data for 2 h. Sleeve opening incidents are highlighted in purple.

The Guidehawk^©^ data comprise a multivariate time series collected over 3 days with a granularity of the minute level for each fracking operation. Initially, variables such as coil and annular pressure (Pa), strain (N), shock (g), temperature (C), and torque (Nm) were recorded at approximately 1,000 samples per second. However, to reduce data volume, these readings were later compressed by considering the maximum value and consolidating them into 1 sample per second. Labeling sleeve incidents using Guidehawk^©^ data is more convenient than using Echo^©^ data, as it captures downhole events by recording six features. In this project, expert knowledge was used to label the data as open and close incidents using two variables: shock and annular pressure. This manual labeling process covered 11 fracking operations, encompassing nearly 2 million data points. Figure 1 illustrates Guidehawk^©^ data with shock and annular pressure (annP).

The sudden jump in pressure accompanied by a spike in shock serve as key features to identify sleeve opening during labeling. The purpose of introducing Guidehawk data in this project is twofold:

- Training Machine Learning model that can identify sleeve incidents after fracking jobs are finished. This helps engineers can easily go through data and check for any unexpected situations instead of manually going through each data point to find sleeve incidents.
- Labeling Echo data becomes challenging when pinpointing the exact time of a sleeve incident using only shock values. However, we can address this issue by utilizing the Guidehawk model and data, as both record the same event at the same timestamp, allowing us to generate accurate labels for the Echo data.

#### 3.1.2. Echo^©^ data

**Figure 2:**
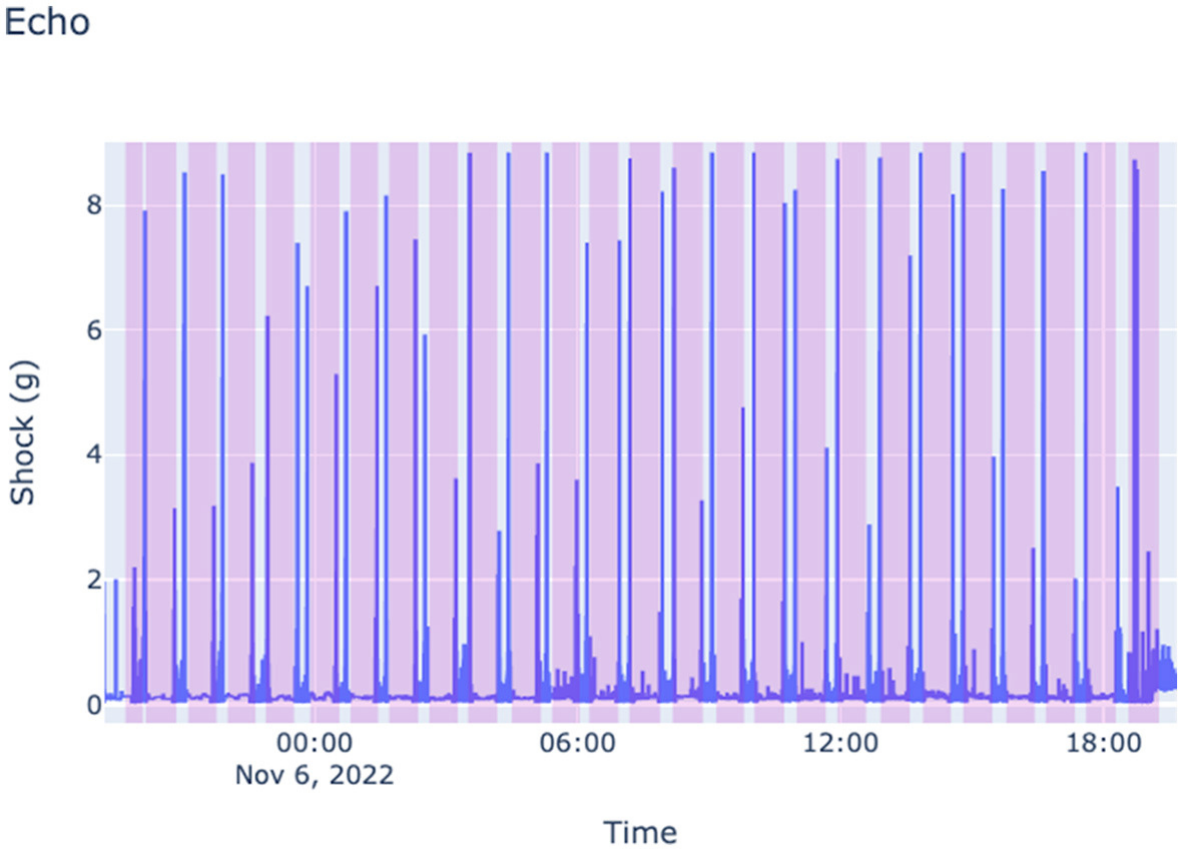
Echo^©^ data for a specific fracking operation. Sleeve opening incidents are highlighted by purple.

Echo^©^ data consist of univariate time series collected in three different orthogonal axes, representing the three-dimensional Cartesian coordinates (x, y, z) on the surface. These three values are then converted into the magnitude of a vector by calculating the geometric values of those axes. The original frequency of the Echo^©^ data is 3,000 Hz. However, we down-sampled it using the average method to 1 sample per second in order to reduce the noise level in the data set. Figure 2 illustrates Echo^©^ data for a specific job.

One particular challenge is to label the Echo^©^ data as it is difficult to pinpoint the exact time of a sleeve incident using only shock values. Thus, we labeled Echo^©^ data by generating labels using guidehawk data. In addition, field reports from fifteen fracking operations were employed for labeling purposes in this project. However, it is crucial to acknowledge that field reports may not always accurately represent the actual events since they record multiple attempts to open the sleeve as one event leading to noisy labeling. Field reports consist of manual entries detailing each well's fracking events. Two essential columns in these reports are the fracking start and end times, which the fracking operator inputs during the operation. An alternative approach to labeling Echo^©^ data involves using Guidehawk^©^ data labels and mapping those labels to Echo^©^ data based on the timestamp in both datasets. This method can result in more accurate labeling. However, it requires having both Echo^©^ and Guidehawk^©^ data for the same fracking operation, which may not always be available. In this project, we did not have Echo^©^ and Guidehawk^©^ data available for the same fracking operation.

#### 3.1.3. Data collection challenges

Location and well type can significantly influence data generalizability. Well type, according to the depth of wells, can be classified as deep or shallow wells. We conducted a two-sample Anderson-Darling test to investigate the potential impact of well depth on data distribution, examining shock values for both deep and shallow wells. This statistical test allows us to evaluate the null hypothesis that both samples are derived from the same population, without needing to define the distribution function of that population. The test results led to the rejection of the null hypothesis at the 0.001% level, indicating that the shock distributions for deep and shallow wells do not originate from the same distribution. This conclusion was drawn because the returned test value exceeded the critical value of 0.1% (6.546). To mitigate this impact during data collection, we diversified our data sources, gathering data from both deep and shallow types of wells, thus minimizing the batch effect. This strategy diminishes the influence of well-type-specific biases.

### 3.2. Data preprocessing

Proper processing is essential to extract meaningful insights from the data. The first in data preprocessing for GuideHawk and Echo data is to ensure that there are no missing values. Missing or incorrect values in time-series data, especially for longer horizons (i.e., 1 h), can significantly impact the entire analysis. Some fracking operations were missing over 10% of the Echo^©^ data—these fracking operations were removed from our data set. To ensure data accuracy, we clipped values within the range of the minimum and maximum values for our sensors. These sensors record downhole events in binary format, which are then converted to float values using Kobold's software. However, in extreme cases, the conversion process can result in values that are outside our sensors' range, such as NaN or very large float values. In this project, we implemented value clipping based on the sensors' range to facilitate the conversion process. In addition, we normalize the data to reduce the impact of different scales for each feature in our dataset. We achieved this by converting the data to a normal distribution.

To prepare both Guidehawk^©^ and Echo^©^ data for time series prediction using a machine learning model, we used a window-based sampling method with overlapping sliding windows, considering all timestamps. The overlapping sliding window, with a window size of *w*_*sample*_, segments the full time-series data stream end-to-end. Once we converted the data into overlapping sliding windows, we split them into separate train and test sets for both Guidehawk^©^ and Echo^©^ data. This allowed us to test the model's performance on unseen data and prevent the unintentional inclusion of future information or data from the target variable into the training process, which can lead to unrealistic model performance and inaccurate prediction. We ensured that the splitting was performed at the operation level to avoid data leakage, where a specific number of fracking operations were randomly selected for the training set (X), while a different number of operations were selected for the test set (Y). This approach effectively eliminates any data point from a fracking operation in the test set that was present in the training set, thus preventing data leakage. For the Guidehawk^©^ data, we split them into three sets: the train set, the validation set, and the test set. We performed this random selection process only once, where we chose nine fracking jobs for training the machine learning model and kept one fracking job aside for the validation set. The job in the validation set was used for hyperparameter tuning. Finally, the remaining job was used to test the model's performance on unseen data.

### 3.3. Feature extraction

Feature engineering for time series data can be accomplished considering three aspects: statistical, temporal, and spectral. For this project, we focused on the statistical and temporal aspects of the data. It is also important to ensure that time-series data are stationary for effective feature extraction. To account for variations in standard magnitude values across different thread-lines or times, we calculated the differences between data values and their lag values. This derivative calculation approach helps ensure invariance over time. After computing the derivatives, statistical features were extracted for the Guidehawk^©^ data as follows:

- Min: minimum value.
- Max: maximum value.
- Standard Deviation: square root of variance.
- Median: middle value for sliding window.
- Mean: average value.
- Skewness: the measure of symmetry of distribution.
- Kurtosis: the measure of whether the data are heavy-tailed or light-tailed relative to a normal distribution.
- 1% and 99% quantile: value for 1% and 99% cut point in distribution.

For Echo^©^ data in addition to statistical features, temporal features are also calculated, which are as follows:

- Total energy: the total energy of the signal.
- Peak to peak distance: the peak to peak distance.
- Entropy: the entropy of the signal using the Shannon Entropy.
- Area under the curve: The area under the curve of the signal computed with trapezoid rule.
- Auto-correlation: autocorrelation of the signal.
- Centroid: the centroid along the time axis.
- Negative and positive turning: number of positive and negative turning points of the signal.
- Distance: computes signal traveled distance.
- Zero cross: computes Zero-crossing rate of the signal.

### 3.4. Machine learning models

#### 3.4.1. Hidden Markov model

The Hidden Markov Model (HMM) (Ghassempour et al., 2014) is a probabilistic approach used to model real-world phenomena that follow the Markov property. The Hidden Markov Model serves as an innovative approach for clustering data by considering both the underlying probabilistic relationships within the data set and the hidden states that generate observable patterns (Ghassempour et al., 2014).

Fracking operations in this project happen in different steps, like releasing BHA, moving to the next sleeve, opening the sleeve, and actual fracking. By using HMM clustering, we can find more information than we can with regular methods. One useful thing we can find is how likely each time period is to be in each group. This helps us know which fracking step is happening in a certain data point. This extra information can help us understand how fracking steps happen one after another.

First, we train the HMM in the training group using a library called hmmlearn. We choose a hidden Markov model with Gaussian distribution because our data looks like a normal distribution, especially the Echo data. We use the Expectation-Maximization (EM) way. We start by guessing model settings (like how likely each step is, how they change, and what the average and spread are) randomly, along with some real examples. While we train, the model changes these settings to match the real examples. We train this Hidden Markov Model for 100 tries.

To find the best number of groups, we use the elbow method. This helps us pick a good number of groups, and we will talk more about it in the Section 4. After we train the Hidden Markov Model using the training data, we obtain the probabilities that each data point belongs to each group. These probabilities are added as additional features when we train the XGBoost model for Echo data.

#### 3.4.2. XGBoost

XGBoost is a machine learning algorithm that uses sequential decision trees and gradient boosting to predict data classes (Ji et al., 2019; Yang et al., 2019). It has demonstrated outstanding performance in predicting structured data in recent years (Wu et al., 2021).

However, in time-series data, XGBoost requires performing feature engineering on feature vectors before training. Before training XGBoost, we conducted feature extraction to capture temporal and statistical relationships within each sliding window. Subsequently, we trained XGBoost using the extracted features, enabling the model to recognize data patterns based on provided labels. Essentially, XGBoost aims to uncover data patterns by utilizing the information derived from the extracted features.

The XGBoost model assesses features obtained from the feature engineering stage and arranges them in order of their capability to distinguish instances of sleeve opening incidents. This procedure empowers the model to concentrate on the most pertinent features, thus enhancing its predictive performance. To address the issue of overfitting and determine optimal hyperparameters for the XGBoost model (such as the number of trees, maximum tree depth, subsampling and column sampling ratios, and learning rate), we employed Bayesian search in combination with cross-validation using the scikit-optimize library.

For the cross-validation process, we opted for 10 fracking jobs out of 11, each time training XGBoost on 9 of the fracking jobs and evaluating its performance on the remaining one. The utilization of Bayesian search with cross-validation allows us to identify the most suitable combination of hyperparameters with a reduced risk of overfitting. The final best hyperparameters comprise 100 trees, a maximum depth of 6, subsample and column sampling ratios of 0.7 each, and a learning rate of 0.1.

#### 3.4.3. LSTM-FCN

The LSTM-FCN (Karim et al., 2018) algorithm uses separate CNN and LSTM components. The CNN components extract local and Hierarchical features, while the LSTM component capturing long-term dependencies from the data. These features are then concatenated and passed through a dense layer with a softmax activation function to determine the probability that the input belongs to class 1 (sleeve opening incident) or class 0 (sleeve is closing incident). The advantage of using LSTM-FCN lies in its ability to capture both higher-level temporal patterns (e.g., delayed effects of strain on detecting sleeve incidents) and low-level temporal patterns (e.g., spikes on shock). This hierarchical learning significantly improves the model's capability to distinguish between different classes in the time series.

The input to the LSTM-FCN is $X\in {\mathbb{R}}^{n_{s}\times w_{s}\times n_{d}}$, where *n*_*d*_ indicates the number of features for data, *n*_*s*_ indicates the number of samples, and *w*_*s*_ is the window size. The LSTM component uses the default sigmoid activation function, while the CNN part consists of three convolution layers with sizes of 128, 256, and 128, followed by global max pooling. In our problem, the LSTM layer is used to independently capture higher-level temporal patterns in the data, taking advantage of its memory capabilities to recognize long-term relationships, such as trends and global peaks in time series. Meanwhile, CNN layers are used to detect low-level temporal patterns, such as sudden changes in values. By combining the strengths of both CNNs and LSTMs, the resulting LSTM-FCN model is capable of capturing both short-term and long-term temporal patterns in the data, making it a popular choice for time series classification tasks.

We extensively delve into the meticulous design decisions intrinsic to the LSTM-FCN architecture. Employing a univariate LSTM-FCN model, we conducted one-step ahead predictions using Echo data. We mainly focus on two important aspects: finding the right number of layers for the LSTM component and making thoughtful decisions about the kernel sizes in the CNN part. The LSTM-FCN framework excels at combining time-related changes and spatial complexities in data sequences. We discuss the significant effects of adjusting the LSTM layer count on the architecture's predictive power. Through thorough testing, we find a balance that manages model complexity and prediction accuracy. This perspective highlights the importance of deeper LSTM layers in capturing detailed time-related nuances, leading to excellent predictions.

Turning our attention to the CNN part, we use a setup with three successive layers, each having 128, 256, and 128 output channels. These layers are accompanied by kernel sizes of 8, 5, and 3, respectively. These carefully chosen settings help the model extract important features from the input data, enabling it to identify detailed patterns and broader trends. The input data is structured as *b*_*s*_×*f*_*n*_×*l*_*s*_, where *b*_*s*_ is the batch size, *f*_*n*_ is the feature count, and *l*_*s*_ is the sequence length. This format fits well with how the CNN works, allowing for the retention of important sequential relationships.

On the other hand, the LSTM module works with data of dimensions *b*_*s*_×*l*_*s*_×*f*_*n*_, where *b*_*s*_ is the batch size, *f*_*n*_ is the feature count, and *l*_*s*_ is the sequence length. The LSTM part consists of 128 layers, each with a hidden dimension of 128. This matches the output dimensions of the CNN, creating smooth compatibility between the LSTM and CNN segments. This harmony in dimensions enables the easy combination of their outputs in later processing stages.

The peak of the architecture's achievement comes when the LSTM and CNN outputs are merged, passing through a fully connected network with 256 input units. This union captures insights from both time-related and spatial viewpoints, forming a solid basis for future prediction tasks. The final output layer is finely tuned to produce two outputs, aligning perfectly with the desired classification goal. This showcases the architecture's adaptability and usefulness across various applications.

To sum up, our detailed examination of the LSTM-FCN architecture underscores the interplay between LSTM layer counts and CNN kernel sizes. This sheds light on their crucial role in achieving top-notch performance. This understanding enhances the toolkit for shaping the LSTM-FCN architecture, allowing it to uncover intricate patterns in sequential data. This marks the beginning of a new era characterized by enhanced predictive modeling capabilities. Supplementary Figure 1 in the Supplementary material illustrates the LSTM-FCN model architecture in more detail.

#### 3.4.4. Transformers

Transformers are a type of neural network architecture that is particularly well-known for their self-attention mechanism, which allows them to capture complex temporal relationships in data (Vaswani et al., 2017; Wen et al., 2023). The general architecture of Transformers consists of two main blocks: an encoder and a decoder. However, in this project, only the encoder was utilized to derive a representation of the data based on its temporal relationships. For the context of the classification problem, the decoder part was not utilized, as the task involves predicting a single label without the need for generating a sequence of outputs, which is the primary purpose of the decoder in sequence-to-sequence tasks. In our project, the encoder uses a self-attention mechanism to capture temporal relationships in data by learning attention scores between each timestep. These scores indicate the importance of each timestep with respect to the given timestep. In sensor data, the self-attention mechanism can detect different patterns, such as sudden changes in one sensor value or the maximum values for sensors. The output representation of the encoder can be used in the final layer to predict labels.

For the multi-head attention mechanism used in this project, the multi-head attention mechanism enables the model to selectively focus on different parts of the input sequence based on their relevance, facilitating the extraction of meaningful features at various temporal scales. The Gaussian Error Linear Unit (GELU) activation function was chosen based on previous research recommendations (Wen et al., 2023). The input to the transformer model is a tensor $X\in {\mathbb{R}}^{n_{s}\times w_{s}\times n_{d}}$, where *n*_*d*_ indicates the number of features for the data, *n*_*s*_ indicates the number of samples, and *w*_*s*_ is the window size. The output dimension of the transformer model is the same as the input dimension. Global max pooling is used to reduce the output dimension to one. Finally, a dense layer followed by a softmax activation function is applied to compute the probability of belonging to either class 1 (sleeve opening incident) or class 0 (sleeve is closing incident). In this project, Transformers are capable of identifying both low-level and high-level patterns and their relationships concerning sleeve incidents. For instance, through multi-head attention, Transformers can discern distinct patterns, such as spikes in shock values and pressure, and their relevance to constant strain values and temperature.

In line with this project, we adopt a strategic decision to configure the transformer's architecture by aligning the number of attention heads with the number of features, leading us to employ four attention heads. To enable the model to effectively capture temporal information inherent in the time series data, we incorporate positional encoding, an essential element of transformer models. Our experimentation reveals that a scaling factor of 100 for the positional encoding optimally contributes to enhancing the model's performance. Furthermore, we emphasize our parameter tuning for the number of transformer blocks, which we set to four for the current project. Supplementary Figure 2 in the Supplementary material illustrates the transformer model architecture in more detail.

#### 3.4.5. Time-series image encoding

The method of encoding time series data into images (Cao et al., 2021) introduces an innovative technique that expands beyond the traditional analysis of 1D signals. Instead, it utilizes Recurrence Plots (RP) to convert time series data into 2D texture images. This novel approach incorporates deep CNN classifiers, leveraging the image representation of time series data. This introduces diverse feature types that were previously unavailable using conventional 1D signal-based methods. The combination of RP and CNN in this method offers new avenues for feature extraction and classification, holding promise for enhancing time series analysis and recognition tasks.

However, a recent study by Elmir et al. (2023) introduces an even more advanced method for visualizing ECG signals, known as the Gramian Angular Field (GAF) technique. Specifically, they propose the Gramian Angular Field Summation (GAF Summation), which surpasses the capabilities of Recurrence Plots (RP) in analyzing time series data. While RP generates a 2D representation of temporal dynamics using a binary matrix to show the recurrence of similar states, GAF Summation provides a more comprehensive and insightful approach.

GAF Summation offers a superior alternative by capturing intricate temporal relationships through the construction of a Gramian matrix. This matrix retains angular distances between data points, presenting a more detailed representation of patterns compared to the binary nature of RP. By harnessing GAF Summation, a deeper and more nuanced understanding of complex temporal sequences can be achieved.

Furthermore, GAF Summation excels in revealing intricate dependencies and non-linearities inherent in time series data. In contrast to RP, which might miss finer structures due to its binary nature, GAF Summation maintains the continuous nature of data relationships. This enhanced capability enables GAF Summation to detect and highlight subtle patterns, periodic behaviors, and non-linear trends, thereby empowering analysts to uncover more profound insights within the data.

**Figure 3:**
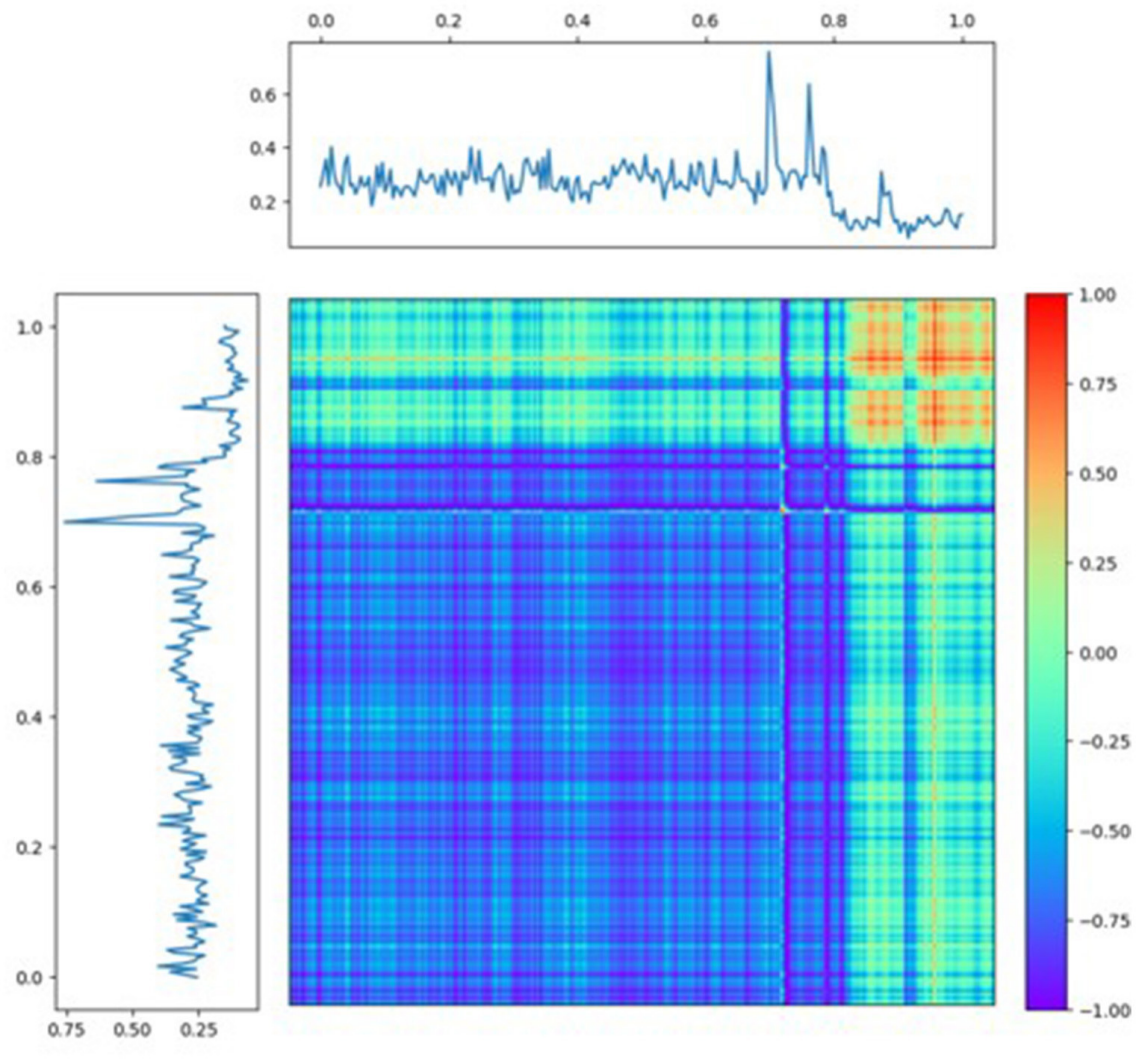
Sample of recurrence plot: converting time series to image.

Our methodology pioneers this transformative approach, utilizing GAF Summation to convert raw time series data into an easily interpretable visual format. By tapping into GAF Summation's capacity to unravel complex temporal dependencies, we access a superior tool for identifying patterns and trends. This goes beyond the capabilities of conventional techniques such as RP, ultimately revealing information that would otherwise remain concealed. Figure 3 provides a visual representation of sample Echo data transformed into an image using the GAF Summation technique. In the resulting image, the two phases of the time series following a sudden decrease are distinguished by the presence of blue and red colors.

Our methodology proceeds by exploiting the strengths of this visually derived information. Each transformed recurrence plot, functioning as an image, is then fed into a ResNet50 architecture, a robust convolutional neural network designed for image classification tasks. This integration is particularly innovative as it capitalizes on the ResNet50's remarkable ability to recognize intricate and abstract patterns within images, which, in this context, are the visual patterns extracted from the GAF Summation technique. Supplementary Figure 3 illustrates a sample of a batch of images to ResNet50.

An essential aspect of our strategy involves the complete training of all the weights within the ResNet50 architecture. This comprehensive training enables the network to adapt and learn intricate features, correlations, and temporal dependencies present within the transformed recurrence plot images. The combined strength of GAF Summation and the ResNet50 architecture contributes to a holistic and complementary approach, effectively addressing the complexities of time series classification tasks.

### 3.5. Proposed framework

In this project, we propose two frameworks: one for Guidehawk^©^ data (multivariate time series) and one for Echo^©^ data (univariate time series data). Using HMM clustering for Echo^©^ data to extract features related to stages of fracking jobs can improve model training, as it captures the relationship between stages in events, especially since the raw data has only one feature like Echo with shock value.

#### 3.5.1. Guidehawk^©^ model

**Figure 4:**
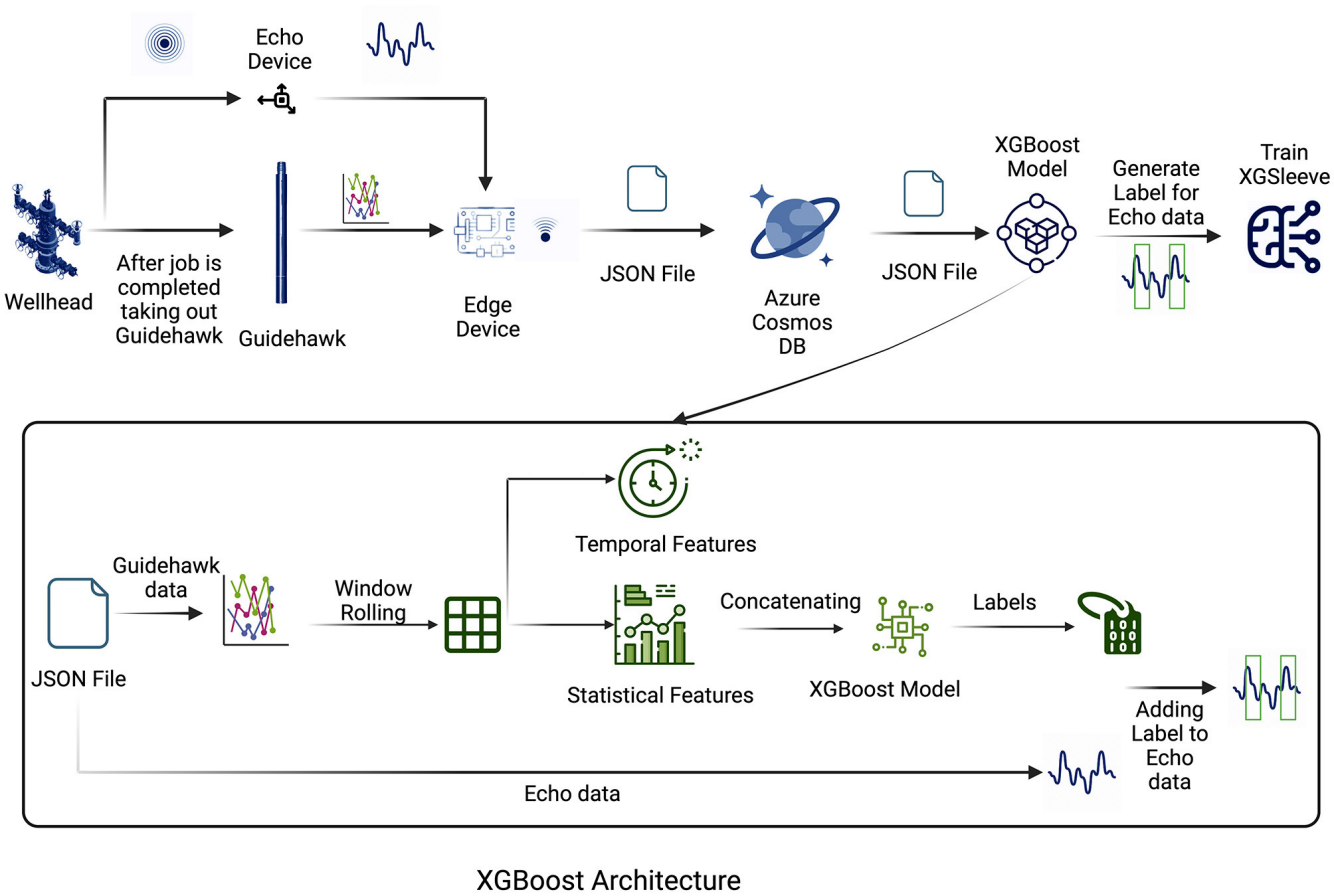
Flowchart of ML model for Guidehawk^©^ data.

The proposed framework for analyzing Guidehawk^©^ data, as depicted in Figure 4, aims to train an XGBoost model for the identification of sleeve opening incidents. The process commences by collecting data from both GuideHawk and Echo sources subsequent to the completion of fracking operations.

GuideHawk data, encompassing coil and annular pressure, strain, shock, temperature, and torque, along with Echo's surface vibration (shock) data, is gathered and transferred to an edge device. This edge device compiles the collected data into a structured JSON file, arranging it chronologically based on timestamps. Subsequently, this JSON file is uploaded to Cosmos DB, serving as a repository for data originating from diverse fracking jobs conducted across Canada.

Within the cloud environment, Guidehawk data undergoes extraction and subsequent feature engineering procedures. Temporal and statistical features are extracted to create an enriched dataset. The XGBoost model, initially trained using GuideHawk data, then takes on the role of generating labels for new fracking jobs. These generated labels are integrated with their respective timestamps. The final dataset incorporates both these appended labels and the corresponding Echo data.

Concluding this process, the XGSleeve model is trained using this enhanced dataset. The aim is to augment the predictive capabilities for upcoming fracking jobs, further enhancing the understanding and forecasting of sleeve opening incidents.

#### 3.5.2. XGSleeve model for Echo^©^ data

**Figure 5:**
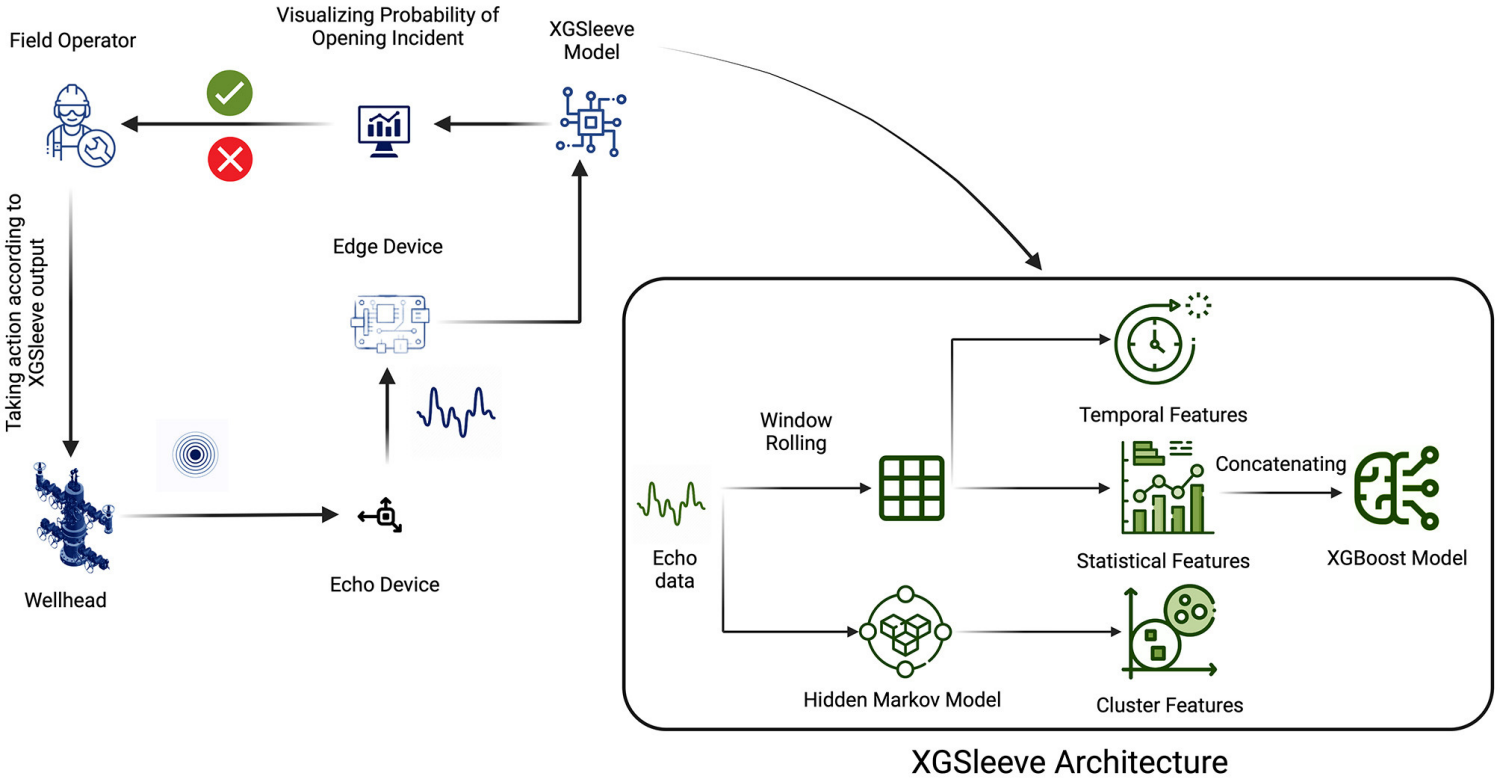
Flowchart of ML model for Echo^©^ data.

After the completion of XGSleeve model training, its deployment in the field offers real-time support to field operators in making informed decisions. The process begins with continuous recording of shock values on the wellhead by Echo data, which is then downsampled to one sample per second. This data is subsequently transmitted to an edge device for the prediction of the ML model, as depicted in Figure 5.

The XGSleeve model functions on the edge device, beginning with the utilization of the pre-trained hidden Markov model to derive cluster probabilities for each timestamp. These probabilities then serve as inputs for the subsequent XGBoost model. Subsequently, temporal and statistical features are extracted. In the subsequent phase of feature engineering, the XGBoost model assesses the present timestamp using the extracted features, thereby producing probabilities that are linked with each occurrence of opening and closing incidents.

These probabilities are then relayed to the operator's monitoring device, facilitating real-time visualization of the ongoing situation. Empowered with these insights, the operator gains the ability to make well-informed decisions, whether to reattempt the opening procedure or proceed to the subsequent steps of the operation. The integration of the XGSleeve model into field operations significantly bolsters efficiency and safety, offering crucial assistance for swift and accurate decision-making during critical fracking procedures.

## 4. Results

This section evaluates our proposed machine learning model on the test data set for both Guidehawk^©^ and Echo^©^ data. Specifically, in this project, we evaluate the model's performance using commonly used metrics such as F1 score, Precision, and Recall. These metrics provide valuable insights into the classification model's effectiveness and are widely employed to assess its accuracy and ability to correctly identify positive and negative instances within the dataset. Additionally, we compare our model's performance with that of several baseline models to demonstrate its effectiveness in addressing the problem at hand. Overall, the results suggest that the XGBoost model is a promising solution for both Guidehawk^©^ and Echo^©^ data.

### 4.1. Guidehawk use case

We used three different models to analyze the Guidehawk^©^ data: XGBoost, Bagging SVM (Baseline Model), and Transformers. We selected these models based on their demonstrated effectiveness in handling multivariate time series data.

#### 4.1.1. Window and derivative size effects on the performance of the XGBoost model

**Figure 6:**
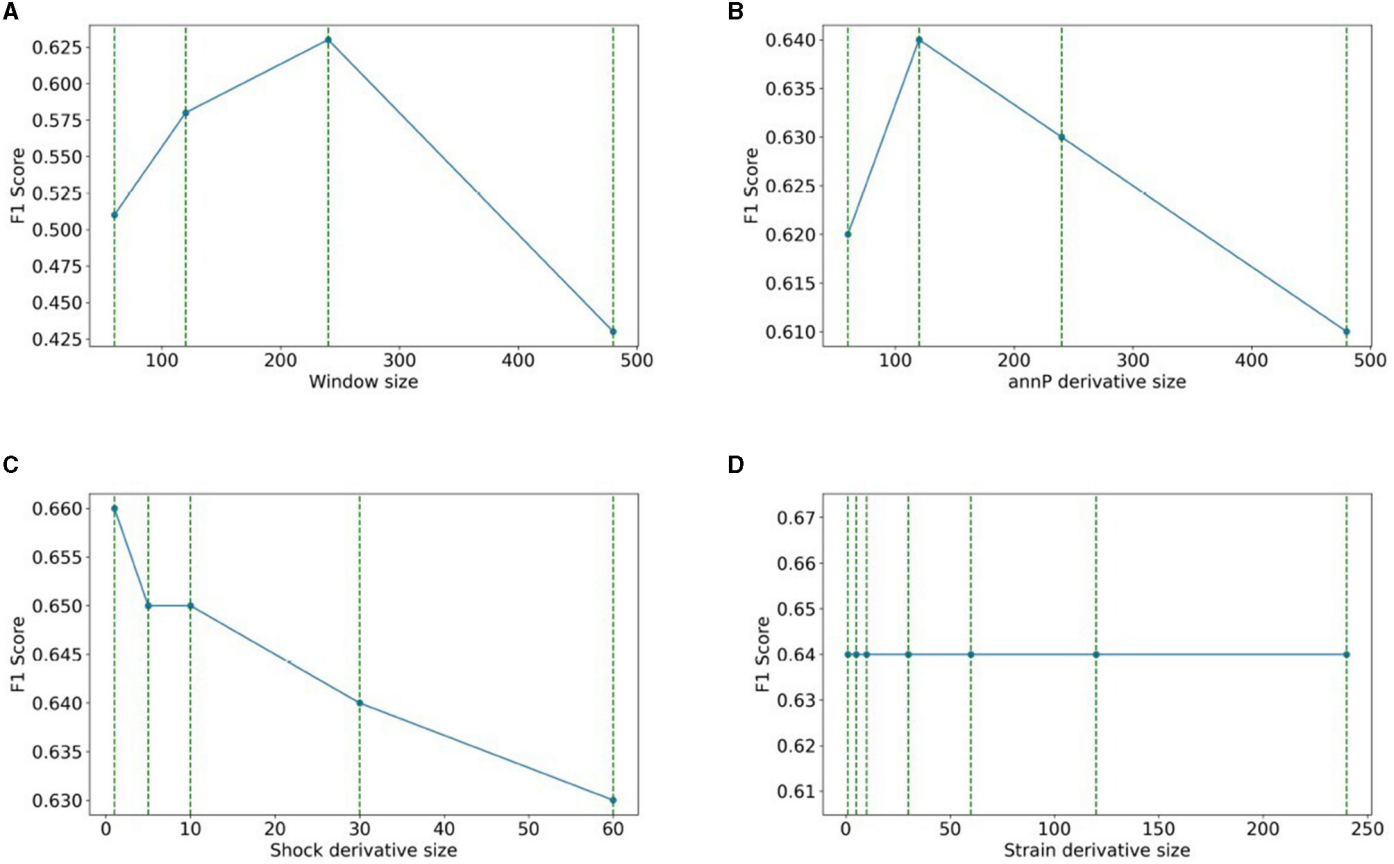
Window and derivative size on the performance of the XGBoost model: **(A)** windows size, **(B)** annP derivative size, **(C)** shock derivative size, **(D)** strain derivative size.

This section explores the impact of window size on the performance of the XGBoost model and the determination of optimal derivative sizes for shock, strain, and annP. We evaluated four window sizes, finding that a window size of 240 yielded the highest F1 score. Our investigations encompassed three experiments to identify the optimal derivative size for each variable. Specifically, annP exhibited optimal performance with a derivative size of 240. Notably, strain's performance remained unaffected by derivative size variations. Conversely, for shock, the most effective derivative size was found to be 1 s, aligned with the rapid changes observed in shock values within that time frame. The summarized results for annP, shock, and strain derivative sizes, along with insightful visualizations, are consolidated in Figure 6, furnishing a comprehensive perspective on our experimental performance and findings.

#### 4.1.2. Comparison of XGBoost model with other models

Table 1 compares the XGBoost, Bagging SVM, and Transformers models for a 240-s sliding window using Guidehawk^©^ data. The XGBoost model outperformed the other models with a 60% F1 score. Training the XGBoost model on an NVIDIA RTX A6000 took less than 5 min, and only two microseconds were required to predict the probability of a new batch sample.

**Table 1:** F1 score, Recall, and Precision for Guidehawk^©^ data.

**Model name**	**F1 score**	**Recall**	**Precision**	**Training time (minute)**
Bagging SVM	0.46	0.53	0.4	45
Transformers	0.52	0.59	0.46	480
**XGBoost**	**0.6**	**0.64**	**0.56**	**5**

The bold values indicate emphasizing the best model result.

**Figure 7:**
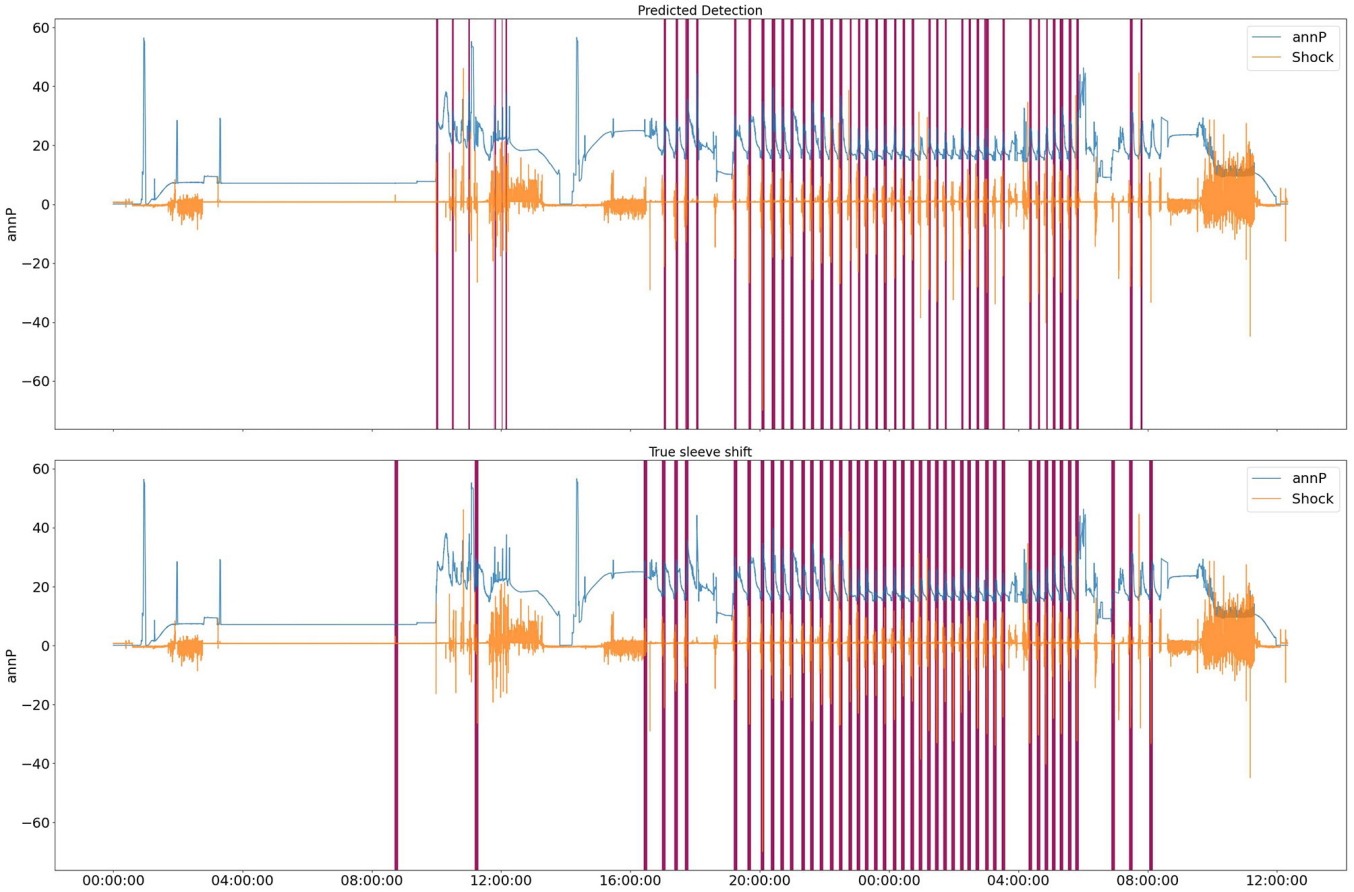
XGBoost predicted labels for test data. Purple parts are labels for a specific range of time.

After plotting the results and comparing the true labels with the predicted ones, we realized that the current performance metric only considers how well the model predicts the labels for each point. However, the objective of this project is to find the time range for the sleeve opening incident. Therefore, we changed our performance metric to a range detection metric. Tatbul et al. (2018) recommended using the F1 score metric for range detection in time series by considering the importance of each point in the range. In our case, the first point in the range is very important, and we assigned a weight of 1.0 to that point, while the remaining points were given equal weight. With the introduction of the new performance metric, we calculated the range F1 score only for our previously top-performing model, XGBoost, which achieved a commendable range F1 score of 0.87. Figure 7 shows the predicted labels and true labels for the test data set.

**Figure 8:**
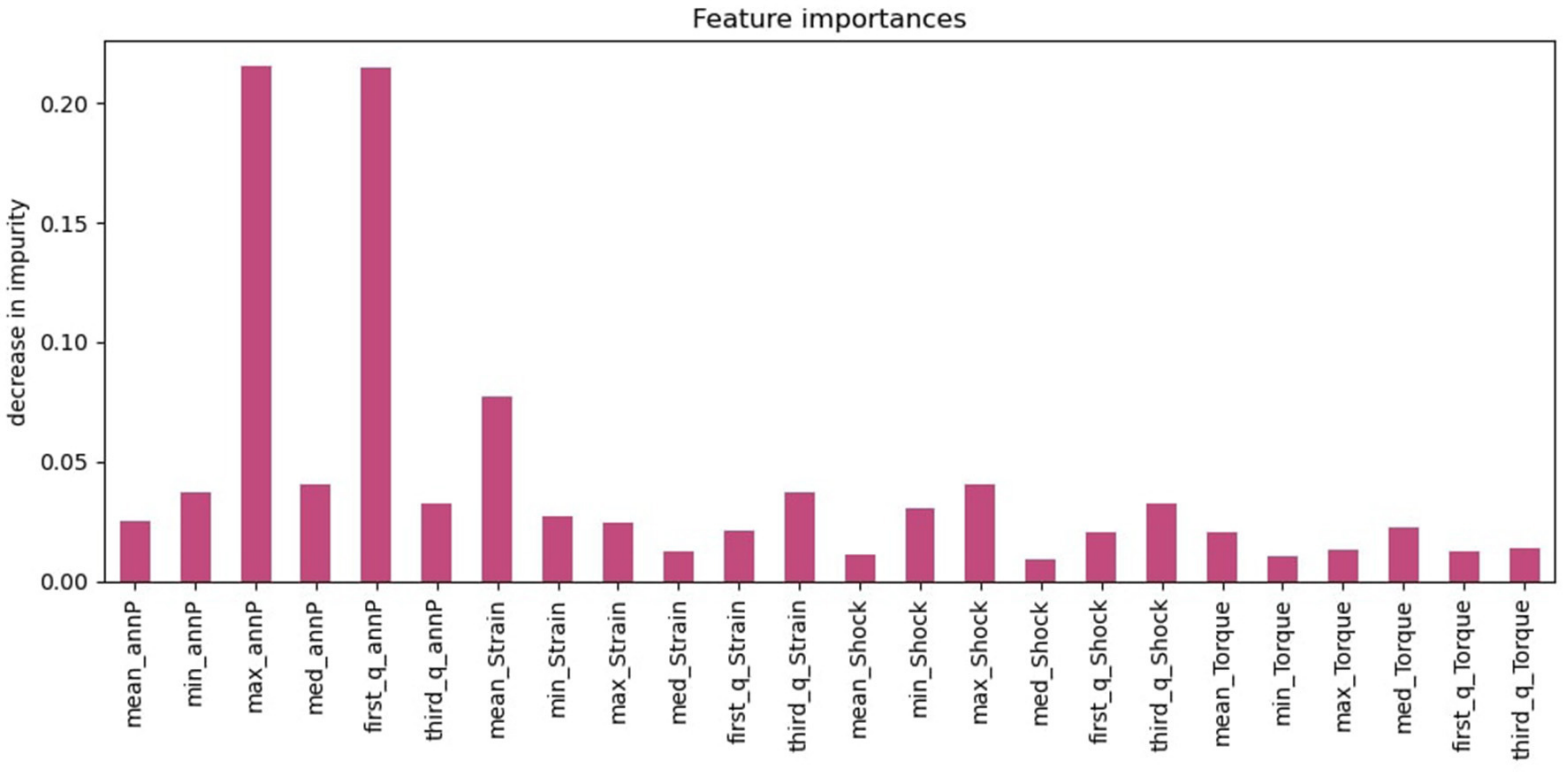
XGBoost feature importance.

Figure 8 depicts the feature importance of the XGBoost model, with the y-axis indicating the decrease in the impurity of each feature. The annular pressure has a significantly higher importance score than the other features—it has the highest decrease in impurity and it results in more pure branching in XGboost. While the annular pressure is the most crucial feature, other features such as Strain and Shock values also predict the outcome.

### 4.2. Echo^©^ use case

To train models with Echo^©^ data, we employed the same window size for models that we experiment with in Guidehawk^©^ data and evaluated models with F1 scores. It is worth mentioning that the range F1 score may not be an appropriate performance metric for Echo^©^ data. Our interest leans more toward accurately predicting each timestep, rather than merely predicting a range of timesteps.

As part of this project, we propose a novel model that combines Hidden Markov Model (HMM) and XGBoost to enhance XGBoost's performance (XGSleeve). HMM is a clustering method used for analyzing sequence data. The underlying assumption of HMM is that the system being analyzed follows a Markov process. In our project, the completion process of sleeve opening involves a cycling process that follows a Markov process and goes through a finite number of stages. By incorporating HMM with XGBoost, we aim to capture the sequential patterns of the data more effectively, leading to improved performance. We trained HMM with different numbers of clusters (1 – 9) to find the appropriate number of clusters.

**Figure 9:**
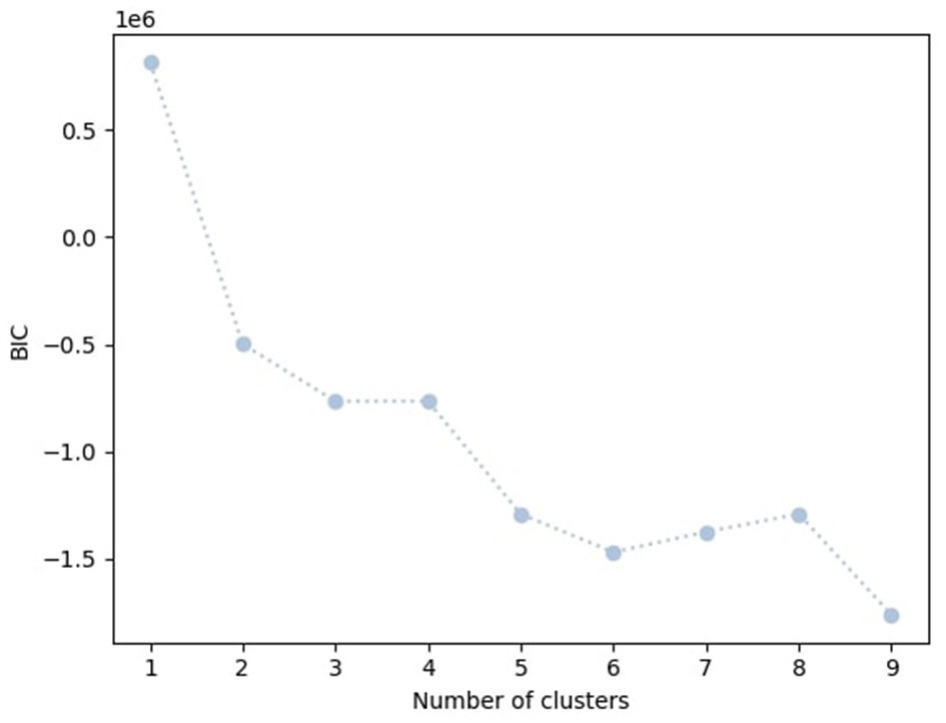
BIC value for different cluster number.

The elbow method is a technique used to determine the reasonable number of clusters for clustering algorithms. It works by plotting the Bayesian information criterion (BIC) value against the number of clusters. As the number of clusters increases, the BIC value decreases. The elbow point is the point at which the rate of decrease in the BIC starts to diminish significantly, resembling an “elbow" in the curve. This point represents a reasonable trade-off between having too few clusters (which might not capture the underlying structure of the data) and having too many clusters (which might overfit the data). Figure 9 illustrates the BIC value for each number of clusters. We can observe that after the fifth cluster, the BIC value fluctuates around a fixed point, and there is not a significant drop in the BIC value.

**Figure 10:**
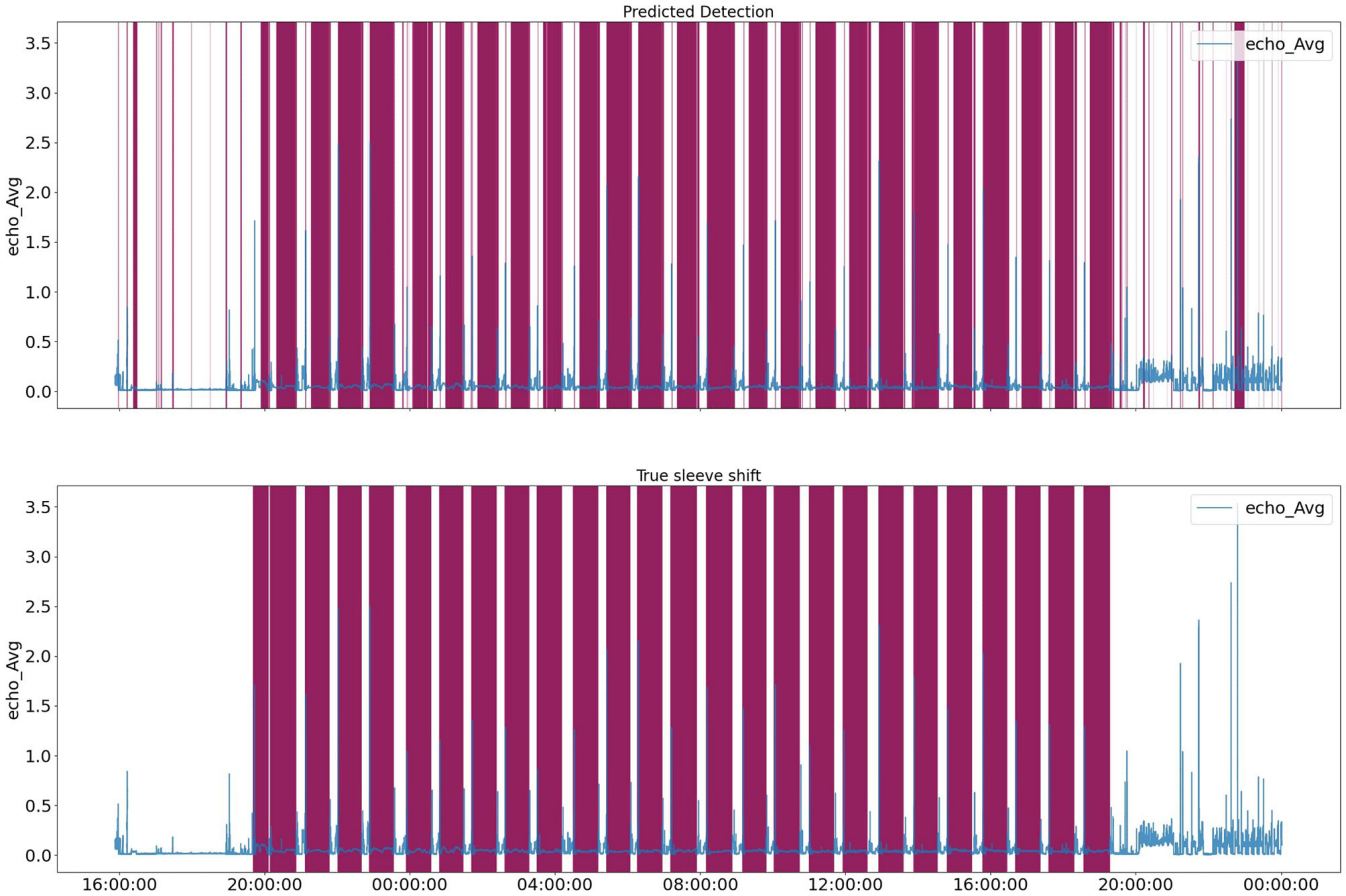
HMM+XGBoost predicted labels for test data. Purple parts are labels for a specific range of time.

Based on the elbow method, the figure suggests that the reasonable number of clusters is 5. We then trained an HMM model with 5 clusters and used the resulting probabilities for each cluster as an additional feature set in combination with temporal and statistical features. Incorporating clustering information resulted as the additional input to the XGBoost model in a notable improvement in the F1 score. Figure 10 shows the target vs. predicted values for the test data.

**Figure 11:**
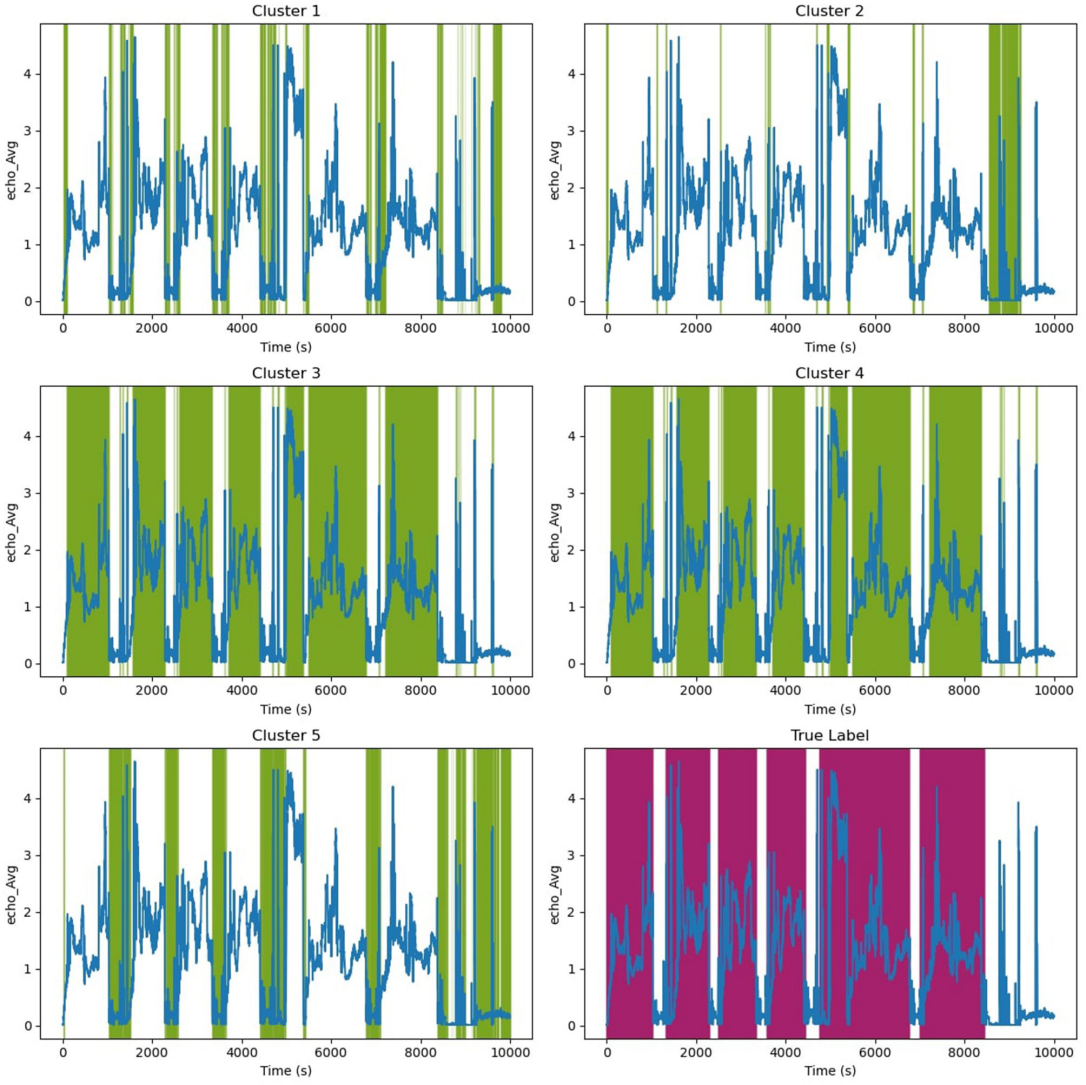
Clusters for specific Echo^©^ singles. Green color highlight clusters.

Figure 11 illustrates HMM cluster for Echo^©^ data.The green color highlights the timesteps that belong to each cluster, while purple highlights the labels. Clusters 3 and 4 may appear identical due to low resolution, but they represent long-term peaks in signal interchangeably. Cluster 5 signifies downtime and stage changes from one well to another. Clusters 2 and 1 represent instances when the tool is releasing pressure to transition to the next well. We can observe that clusters 3 and 4 closely represent the labels, which are highlighted in purple.

**Figure 12:**
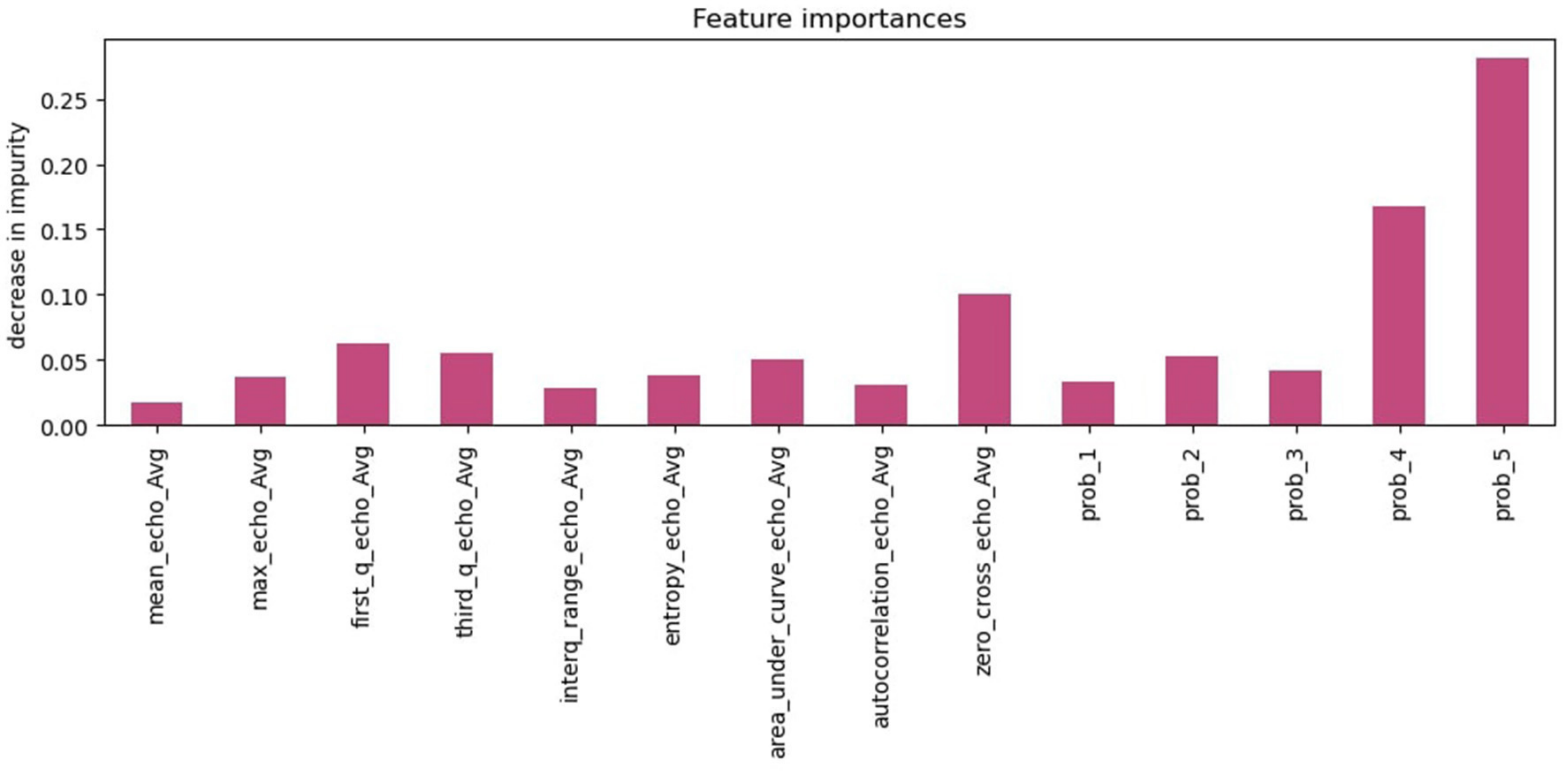
HMM+XGBoost feature importance.

Figure 12 depicts the feature importance of the HMM+ XGBoost model, with the y-axis indicating the decrease in the impurity of each feature. The probability derived from the HMM model is the most important feature, with a significantly higher importance score compared to the other features. While the HMM probability is the most crucial feature, other features such as the number of zero-cross and quantile values also predict the outcome. These features are important because they provide additional information that can help the model differentiate between opening and non-opening incidents.

### 4.3. Comparison of XGSleeve with baseline models

We employed two different models as the baseline models for the Echo^©^ data: XGBoost and LSTM-FCN to compare with our proposed XGSleeve model. Table 2 reports F1-score for both XGBoost and LSTM-FCN models. The table shows that the XGSleeve model significantly outperforms LSTM-FCN and Time-Series Image Encoding models. Moreover, adding HMM features to the data (XGSleeve model) improves F1 score of the XGBoost model by 10%.

**Table 2:** F1 score, Recall, and Precision for Echo^©^ data.

**Model name**	**F1 score**	**Recall**	**Precision**	**Training time (minute)**
**XGSleeve**	**0.87**	**0.86**	**0.89**	**6**
XGBoost	0.77	0.79	0.73	6
LSTM-FCN	0.65	0.68	0.63	300
Time-series image encoding	0.49	0.57	0.53	229

The bold values indicate emphasizing the best model result.

## 5. Discussion

Throughout this project, we have explored the application of various ML methods to predict sleeve opening incidents. The best results were obtained using the XGBoost model for both Echo^©^ and Guidehawk^©^ data. Due to the unique and complex sensor data structure, we placed particular emphasis on feature engineering and window size. We ran several experiments to identify the best hyper-parameters for window size and derivative values.

We employed two distinct ML methodologies to predict sleeve opening incidents. The first technique used a flat feature input, incorporating both historical and current data. XGBoost and HMM + XGBoost models were implemented based on this approach. The second method evaluated the temporal dependencies of features. We employed LSTM-FCN and Transformer models to assess the impact of sequential data structures on prediction. The results indicate that ML models can predict sleeve opening incidents with relatively good accuracy. The XGBoost and HMM + XGBoost model achieved the best performance, with an F1-score of 87% for Guidehawk^©^ and Echo^©^ data, respectively. After conducting several experiments, we have found that the performance of recurrent ML and attention methods, including LSTM and Transformer, was not superior to the flat-feature-based prediction models. This was likely due to the high data requirements needed for training these deep-learning models. Despite the abundance of data points from these five fracking operations, we need to increase the number of fracking operations included in the training data for better generalization in our deep learning model. This is necessary because each fracking operation contributes to learning complex patterns.

During the data collection process, we discovered that collecting data for a specific location leads to overfitting. This can be justified by the fact that wells across Canada differ due to variations in well depth. Our findings indicate that training machine learning models on deep wells cause the XGBoost model to focus heavily on shock values. However, collecting more data from both types of wells results in different feature importances, with annP becoming the more critical factor. For this type of problem, it is crucial to gather data from a diverse range of fracking operations rather than focusing solely on collecting more data from the same fracking operation.

Furthermore, the proposed framework was intended to function as a unified architecture. The initial plan was to train the XGBoost model on Guidehawk^©^ data. The trained XGBoost model would then label the remaining Guidehawk^©^ data without labels in the data warehouse. Subsequently, these labels would be mapped to Echo^©^ data based on timestamps, and the proposed HMM+XGBoost model would be trained on this combined dataset. However, this approach was not implemented as Kobold did not provide both Guidehawk^©^ and Echo^©^ data for the same job. This is because collecting Guidehawk^©^ data is more expensive than Echo^©^ data, and most of their clients do not request its use.

Moving forward, there are three potential directions for further exploration. The first area is related to refining our machine-learning models through further hyperparameter tuning. Hyperparameters can significantly influence the effectiveness of our models, and improved tuning can often result in improved predictive accuracy. This could involve a thorough exploration of various combinations of hyperparameters to identify those that are most suitable for our specific task. This procedure might involve a meticulous adjustment of hyperparameters such as learning rates, regularization values, and the number of trees, which could significantly impact the performance of our models. We would also need to consider strategies such as k-fold cross-validation to ensure that the results are generalizable and robust.

The second area is the reconsideration of the current data labeling approach. Given that noisy labels can significantly affect the performance of our models, relabeling the data could be a useful step to reduce such noise levels. We can investigate various noise reduction techniques, which could range from simple filtering methods to more advanced machine learning-based approaches. The refinement of our labels could potentially enhance the performance of our models.

The third area contemplates diving deeper into the data to explore additional data features and domain knowledge. A comprehensive understanding of the domain can often help in identifying valuable features that might be overlooked with a purely algorithmic approach. Additionally, we should consider implementing more advanced machine learning methods such as auto-encoders. These techniques can be particularly useful for complex datasets like ours, as they can learn a compressed representation of the input data, which often leads to more efficient extraction of meaningful features. This approach may allow us to discover and exploit better representations of sensor data, and potentially improve the effectiveness of our models. By focusing on these three areas, we aim to enhance our understanding of the data and our modeling capabilities, potentially leading to more powerful and effective machine-learning solutions.

## Data availability statement

The raw data supporting the conclusions of this article will be made available by the authors, without undue reservation.

## Author contributions

SS and SJ contributed to the conception and design of the model, with SS developing the code and writing the original paper. SJ providing critical feedback, editing the manuscript, and performed a peer review of the paper. DC and PW provided expert opinions and collected data for the project and played a key role in the analysis and interpretation of the results. All authors have read and approved the final manuscript.
