# Supplementary material for: XGSleeve: detecting sleeve incidents in well completion by using XGBoost classifier
Source: Front Artif Intell. 2023 Sep 13;6:1243584. doi: 10.3389/frai.2023.1243584 (PMC10533988; doi:10.3389/frai.2023.1243584)
Supplement: Supplementary file 1 [file Data_Sheet_1.PDF]

Supplementary Material

1 LSTM-FCN

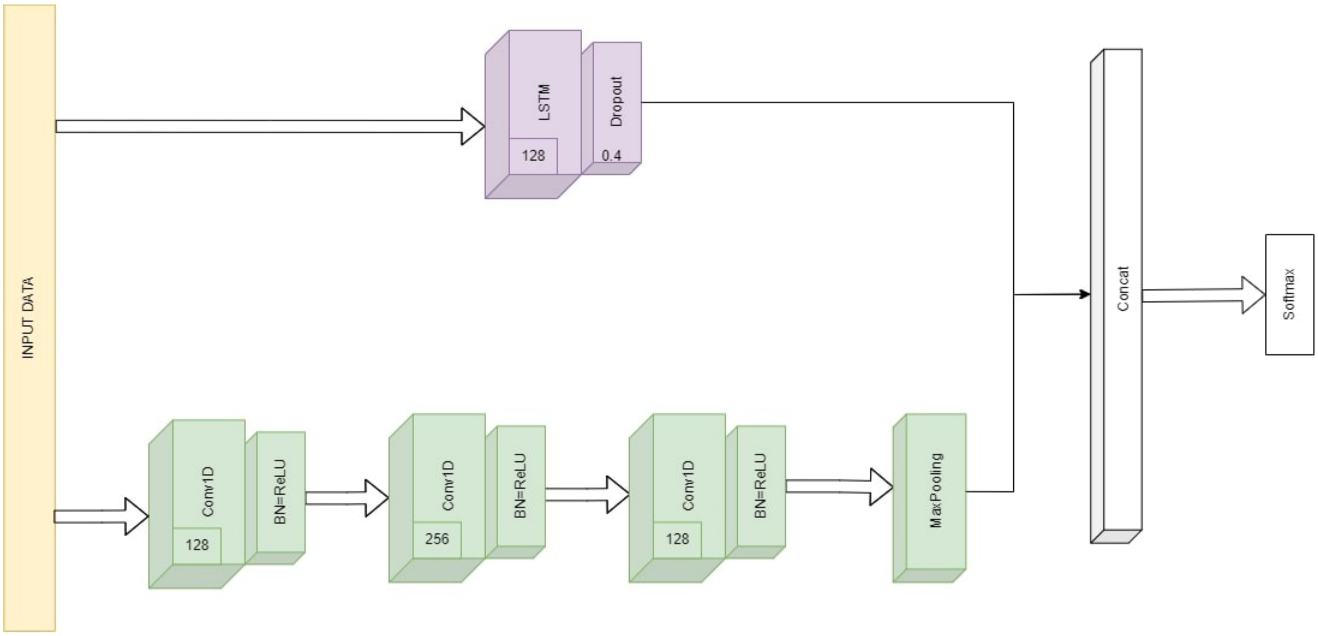

Figure S1. LSTM-FCN Model Architecture

2 TRANSFORMERS

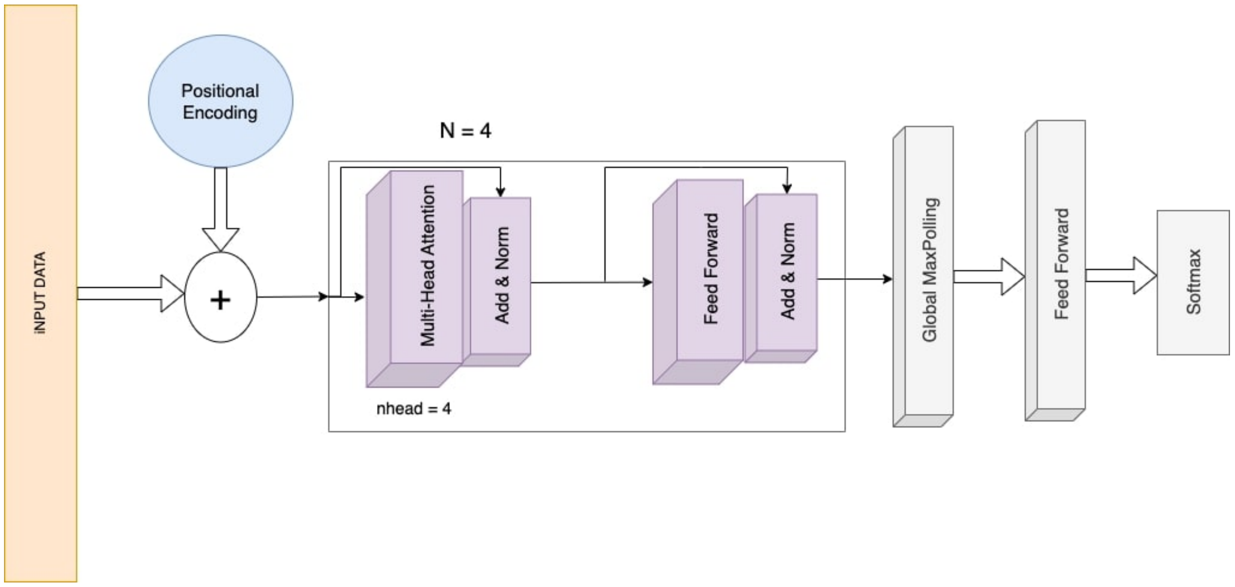

Figure S2. Transformer Model Architecture

3 TIME-SERIES IMAGE ENCODING

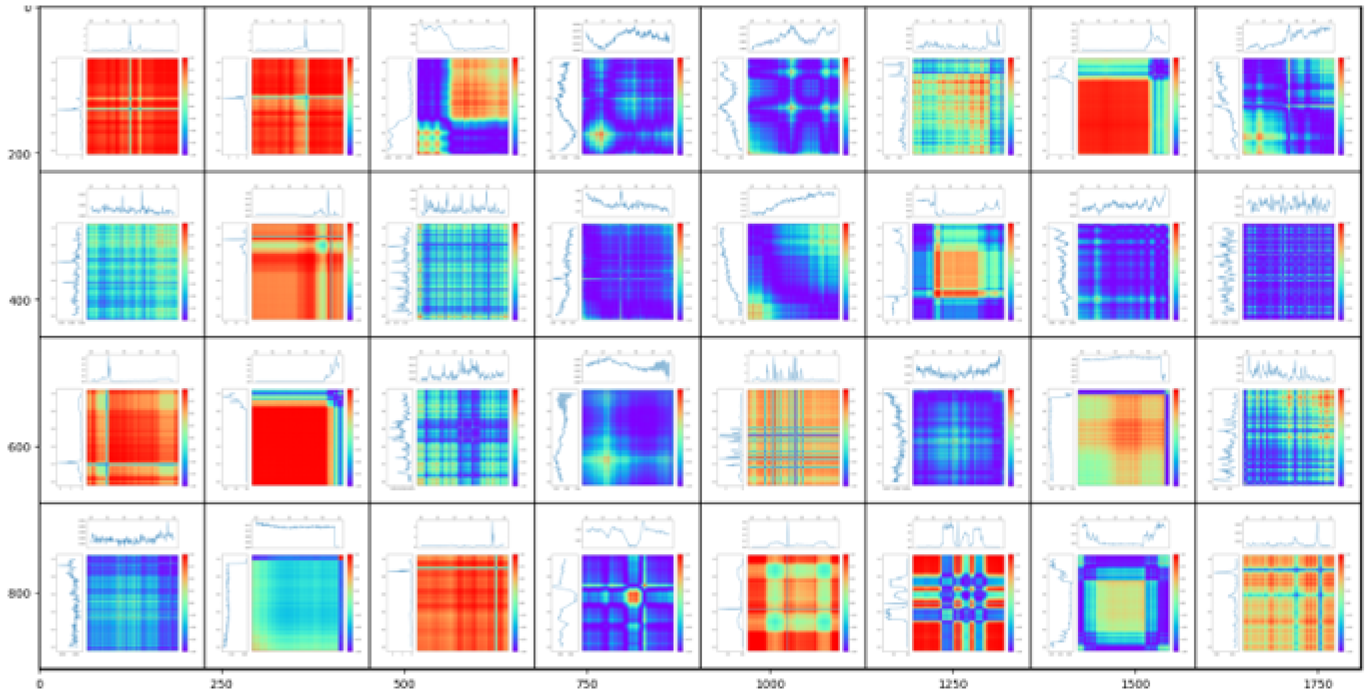

Figure S3. Batch of Time Series Data in Image format

4 PSEUDOCODE FOR XGSLEEVE

Algorithm 1 Training Hidden Markov Model

$N \leftarrow n$ ▷ Number of fracking job in training data
for $i \leftarrow 1, n$ **do**
  $x_n \leftarrow X$ ▷ Get data for a fracking job
  $j \leftarrow 5$ ▷ Set number of clusters
  $Model = RunViterbiAlgorithm(j);$
end for

Algorithm 2 Training XGSleeve

Require: M_{hmm} ▷ Hidden Markov Model
$N \leftarrow n$ ▷ Number of fracking job in training data
for $i \leftarrow 1, n$ **do**
  $x_n \leftarrow X$ ▷ Get data for a fracking job
  $x_{hmm} = M_{hmm}(x_n)$
  $x_{temporal,statistical} \leftarrow x_n$
  $XGSleeve = XGBoost(x_{temporal,statistical}, x_{hmm});$
end for
